# A Rare Malignant Transformation of an Ovarian Cystic Teratoma: A Case Report

**DOI:** 10.1155/2018/6892783

**Published:** 2018-07-09

**Authors:** Manju Rachel Mathew, Anita Ramdas, Susy S. Kurian, Linu Kuruvilla, Neelima Singh

**Affiliations:** ^1^Department of Pathology, Pondicherry Institute of Medical Sciences, Ganapathichettikulam, Kalapet, Puducherry 605014, India; ^2^Department of Radiodiagnosis, Pondicherry Institute of Medical Sciences, Ganapathichettikulam, Kalapet, Puducherry 605014, India; ^3^Department of Obstetrics and Gynecology, Pondicherry Institute of Medical Sciences, Ganapathichettikulam, Kalapet, Puducherry 605014, India

## Abstract

Mature cystic teratoma (MCT) is the commonest germ cell neoplasm of the ovary but malignant transformation is a rare occurrence (1-2%). Of these malignancies documented in literature the commonest are squamous cell carcinoma and adenocarcinoma. Urothelial carcinomas arising in an MCT are a rare occurrence and only 7 cases have been reported in literature. We report a case of an MCT which was complicated by the presence of urothelial carcinoma confirmed on histopathological examination.

## 1. Introduction

According to the 2014 WHO classification, mature cystic teratomas (MCT) constitute approximately 20% of all ovarian neoplasms also being the commonest germ cell tumor [1]. They are tumors which can form mature tissues derived from ectoderm, mesoderm, and endoderm and are usually cystic [2]. The majority of these tumors are benign with malignant transformation being rare and reported in only 1-2% of cases [3, 4]. Among the malignant transformations, squamous cell carcinoma and adenocarcinoma are the most commonly reported [5]. Urothelial carcinomas arising in an MCT is rare [3–9]. This is a report of such a case in a 50-year-old lady.

## 2. Case Report

**Figure 1:**
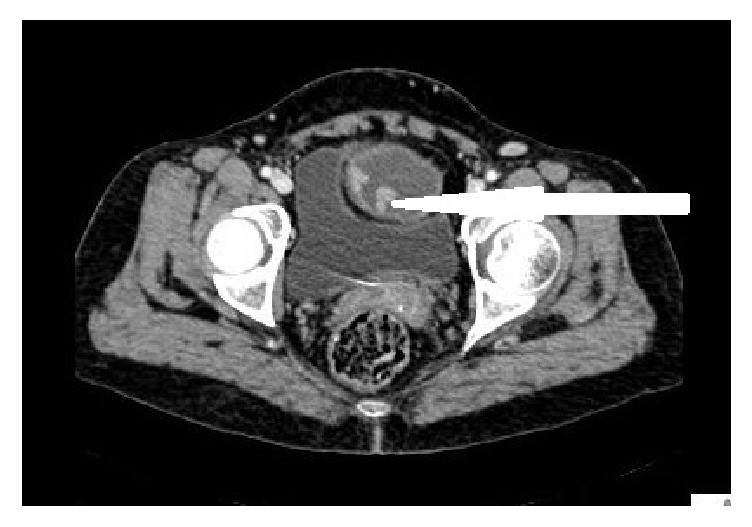
Contrast enhanced computed tomography (CE-CT) image showing cystic right ovary with arrow pointing to enhanced solid densities within the cyst.

A 50-year-old lady presented to the Pondicherry Institute of Medical Sciences Hospital at Puducherry, India, with a complaint of acute abdominal pain. Contrast enhanced computed tomography (CECT) demonstrated the presence of bilateral ovarian mature cystic teratomas. Contrast enhancement within the right ovarian cyst suggested the possibility of malignant transformation (Figure 1). Tumor marker carbohydrate antigen- (CA-) 125 was 27 IU/mL (normal <35 IU/mL). She underwent total abdominal hysterectomy and bilateral salpingo-oophorectomy.

**Figure 2:**
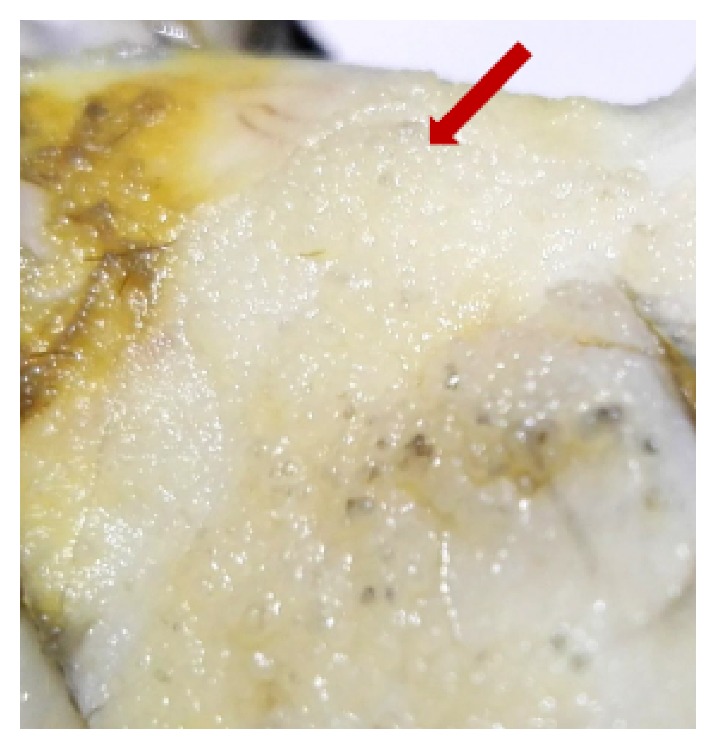
Blunt pale projections on an otherwise smooth cyst wall.

Gross examination showed the right ovary to be cystic and measured 12cms in diameter and is predominantly smooth except for an area of 3cm^2^ which had blunt pale soft projections (Figure 2). The left ovary was grossly normal measuring 3cms in greatest diameter.

**Figure 3:**
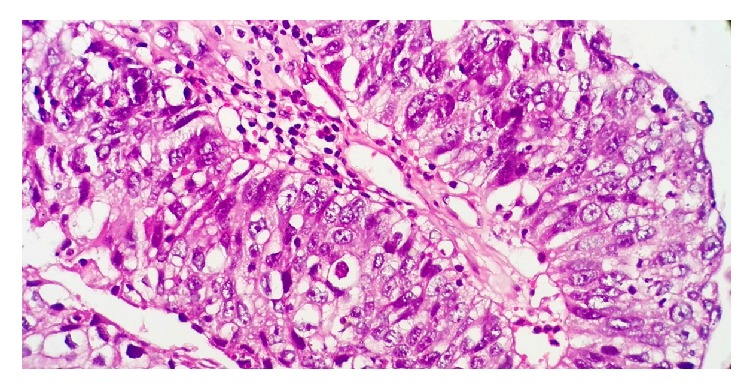
High power view of histomorphology of urothelial carcinoma (H&E 400X magnification).

**Figure 4:**
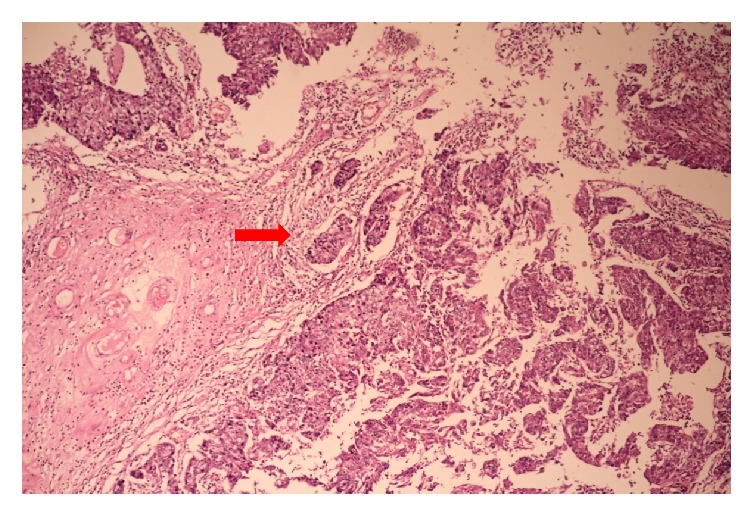
Low power view of urothelial carcinoma with arrow pointing at nests of invasive malignant epithelial cells in ovarian stroma (H&E 10X magnification).

**Figure 5:**
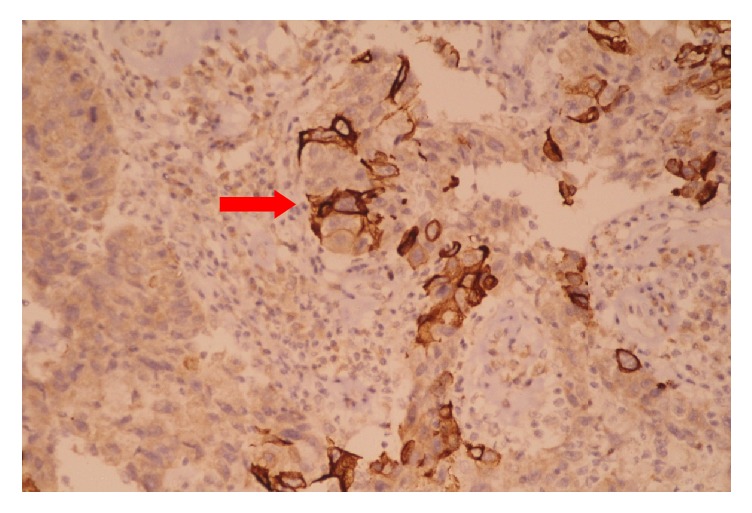
High power view of tumor cells showing cytoplasmic and membranous positivity for Uroplakin II (IHC 40X magnification).

Microscopically the left and right ovary showed various mature tissues including bronchial mucosa, apocrine glands, cartilage, and skin with adnexal structures. The microscopy of the soft pale projections of the right ovary had papillary structures with fibrovascular cores which were lined by transitional epithelium exhibiting nuclear pleomorphism, hyperchromatism, and increased mitotic activity (Figure 3). There was evidence of invasion of the ovarian stroma by nests of malignant epithelial cells (Figure 4). The inked ovarian capsular surface was free of tumor. Immunohistochemistry (IHC) of the urothelial carcinoma showed cytoplasmic and membrane positivity for Uroplakin II (Figure 5). A diagnosis of ovarian cystic teratoma with primary invasive urothelial carcinoma (TNM stage pT1aNxMx) was made based on the Pathological Stage Classification by the American Joint Committee on Cancer (AJCC) 8^th^ edition [10].

The patient was reviewed till 3 months following surgery and follow-up CECT revealed no evidence of recurrent tumor in the abdomen and pelvis.

## 3. Discussion

Urothelial carcinomas complicating an MCT is rare and only 7 such cases have been reported in literature so far [3–9]. The diagnosis of primary invasive urothelial carcinoma in the present case was made by morphology and immunohistochemically demonstrating Uroplakin II in the malignant cells.

A review of the salient features of the 7 prior cases and that of the present patient is presented in Table 1 [3–9].

Studies by Al-Rayyan et al. and Kikkawa et al. showed varying number of patients with malignant transformation of an ovarian MCT who were postmenopausal or premenopausal thus indicating that menopause itself does not pose as a risk factor for malignant transformation in an MCT [4, 11]. Older age group ( >45 years), elevated levels of CA-125 (35 IU/mL), preoperative tumor size (>9.9cm), and characteristic imaging findings were noted as independent risk factors for malignant transformation of an MCT [3, 11, 12]. The present case differed in that no elevation of CA-125 (27 IU/mL) was noted unlike majority of the previous cases [3–9].

The pathogenesis of urothelial carcinoma in an MCT was hypothesized in two studies from Korea which noted the presence of normal urothelium adjacent to the urothelial carcinoma hence suggesting that prolonged and persistent irritation of the urothelium by lipid material induced the secondary carcinomatous change [7, 9].

Lee et al and Dasgupta et al. stated the importance of differentiating the urothelial carcinoma arising in an MCT from a primary transitional cell carcinoma of the ovary (TCC-O) which is a subtype of surface epithelial tumors [6, 7]. Lee stated that the urothelial carcinoma of the urinary tract expressed Uroplakin III but is negative for WT1. On the contrary TCC-O is negative for Uroplakin III and positive for WT1 [7]. Smith et al. and Li et al. in 2014 showed that Uroplakin II is a more sensitive immunohistochemical marker than Uroplakin III for detecting urothelial carcinoma of the urinary tract [13, 14]. In the present case the urothelial carcinoma arising in the MCT showed cytoplasmic and membrane positive staining for Uroplakin II suggesting its origin from the urothelium of the teratoma. The possibility of metastatic deposits from a primary urinary tract carcinoma was ruled out by radiological evaluation showing no lesion in the urinary system. Hence a diagnosis of primary invasive urothelial carcinoma arising in a mature cystic teratoma of the ovary was made.

## 4. Conclusion

Malignant transformation in an MCT is a rare complication but must be suspected in high risk cases of age > 45 years, size larger than 9cm, and elevated tumor marker CA-125 levels. Urothelial carcinomas arising in an MCT of ovary is a rare occurrence and to the best of our knowledge this is the eighth such case reported in literature.

## Figures and Tables

**Table 1:** Reported cases of urothelial carcinoma arising from mature cystic teratoma of ovary.

Study	Age	Menopause	Symptoms	Tumor size (cm)	Laterality	Tumor marker elevated	FIGO stage	Primary surgery	Further Treatment	Follow-up
Lee et al, 1999 [9]	67	Post	Voiding difficulty, lower abdominal pain	14x7x5	Left	CA-125 CA 19-9	IC	TAH+BSO+omentectomy	Chemotherapy; carboplatin, etoposide	NED 5 months

Kido et al, 1999 [5]	48	NA	NA	NA	NA	NA	IC	NA	NA	NDA

Yamaguchi et al, 2007 [3]	48	NA	Abdominal mass	20	Left	CA-125 CEA SCC	IA	TAH+BSO+omentectomy		NED 10y

Rayyan et al, 2009 [4]	45	Pre	Pain, bleeding	8	Left	NA	IA	LSO	Surgical Staging	NED>5 y

Lee & Lee, 2010 [7]	52	Pre	Abdominal mass	22x19x5	Right	NA	IA	RSO	NA	NED 15 mo

Chuang et al, 2015 [8]	54	Post	Abdominal mass	20x13x21	Right	CA-125, CA 19-9	IA	TAH+BSO+omentectomy+BPLA	None	NED 8 mo

Dasgupta et al, 2015 [6]	50	Post	Pain and abdominal swelling	11x9x6	Right	CA-125	IA	TAH+BSO	None	NED

Present case	50	NA	Pain	12x8x5	Right	-	IA	TAH+BSO	None	NED 3 mo

BPLA= bilateral pelvic lymphadenectomy, BSO= bilateral salpingo-oophorectomy, FIGO= International Federation of Obstetrics and Gynecology, LSO= left salpingo-oophorectomy, NA= not available, NDA=no data available, NED=no evidence of disease, RSO= right salpingo-oophorectomy, and TAH= total abdominal hysterectomy.

## Data Availability

The authors declare that the data cited in this report are available in the references mentioned in this paper and can be accessed from Pubmed.

## References

[1] Kurman R. J., Carcangiu M. L., Herrington C. S., Young R. H. (2014). *WHO Classification of Tumours of Female Reproductive Organs*.

[2] Rosai J, Ackerman L. V. (2011). Ovary. *Rosai and Ackermans Surgical Pathology*.

[3] Yamaguchi K., Mandai M., Fukuhara K. (2008). Malignant transformation of mature cystic teratoma of the ovary including three cases occurring during follow-up period. *Oncology Reports*.

[4] Al-Rayyan E. S., Duqoum W. J., Sawalha M. S. (2009). Secondary malignancies in ovarian dermoid cyst. *Saudi Medical Journal*.

[5] Kido A., Togashi K., Konishi I. (1999). Dermoid cysts of the ovary with malignant transformation: MR appearance. *American Journal of Roentgenology*.

[6] Dasgupta S., Bose D., Bhattacharyya N., Biswas P. (2015). Mature cystic teratoma with malignant transformation of teratomatous urothelial cells: Rare case presentation. *Clinical Cancer Investigation Journal*.

[7] Lee O.-J., Lee H.-C. (2010). Urothelial (Transitional Cell) carcinoma arising in mature cystic teratoma: A case report. *The Korean Journal of Pathology*.

[8] Chuang H.-Y., Chen Y.-T., Mac T.-L. (2015). Urothelial carcinoma arising from an ovarian mature cystic teratoma. *Taiwanese Journal of Obstetrics and Gynecology*.

[9] Lee H. H., Shim J. Y., Lee C. (1999). A case of papillary transitional cell carcinoma arising from the benign cystic teratoma of ovary. *Korean Journal of Obstetrics & Gynecology*.

[10] Movahedi-Lankarani S. (2017). Protocol for the examination of specimens from patients with primary tumors of the ovary, fallopian tube or peritoneum. *College of American Pathologists*.

[11] Kikkawa F., Nawa A., Tamakoshi K. (1998). Diagnosis of squamous cell carcinoma arising from mature cystic teratoma of the ovary. *Cancer*.

[12] Stamp G. W. H., McConnell E. M. (1983). Malignancy arising in cystic ovarian teratomas. *BJOG: An International Journal of Obstetrics & Gynaecology*.

[13] Smith S. C., Mohanty S. K., Kunju L. P. (2014). Uroplakin II outperforms uroplakin III in diagnostically challenging settings. *Histopathology*.

[14] Li W., Liang Y., Deavers M. T. (2014). Uroplakin II is a more sensitive immunohistochemical marker than uroplakin III in urothelial carcinoma and its variants. *American Journal of Clinical Pathology*.

